# Evaluation of the Antifungal Activity of the Novel Oral Glucan Synthase Inhibitor SCY-078, Singly and in Combination, for the Treatment of Invasive Aspergillosis

**DOI:** 10.1128/AAC.00244-18

**Published:** 2018-05-25

**Authors:** M. Ghannoum, L. Long, E. L. Larkin, N. Isham, R. Sherif, K. Borroto-Esoda, S. Barat, D. Angulo

**Affiliations:** a Center for Medical Mycology, Case Western Reserve University and University Hospitals Cleveland Medical Center, Cleveland, Ohio, USA; b Scynexis, Inc., Jersey City, New Jersey, USA

**Keywords:** invasive aspergillosis, SCY-078, combination MIC

## Abstract

Invasive aspergillosis remains a major cause of death among the immunocompromised population and those receiving long-term immunosuppressive therapy. In light of increased azole resistance, variable outcomes with existing echinocandin monotherapy and combination therapy, and persistent high mortality rates, new antifungal agents for the treatment of invasive aspergillosis are clearly needed. SCY-078 is the first-in-class triterpenoid antifungal, a novel class of glucan synthase inhibitors with broad *in vitro* and *in vivo* activity against a broad spectrum of Candida and Aspergillus species. *In vitro* testing of clinical strains of Aspergillus fumigatus and non-fumigatus Aspergillus strains showed that SCY-078 had potent fungistatic activity (minimum effective concentration for 90% of strains tested = 0.125 μg/ml) compared with the activities of amphotericin B (MIC_90_ = 8 μg/ml) and voriconazole (MIC_90_ = 2 μg/ml). Testing of SCY-078 in combination with isavuconazole or voriconazole demonstrated synergistic activity against the majority of the azole-susceptible strains tested, and SCY-078 in combination with amphotericin B was synergistic against the azole-susceptible strains, as well as one known resistant *cyp51A* mutant. SCY-078 may be an important additional antifungal for first-line or salvage monotherapy or combination treatment of invasive aspergillosis.

## INTRODUCTION

Aspergillus species are ubiquitous fungi which for the most part are saprophytic. However, these species can be the cause of allergic reactions, including bronchopulmonary aspergillosis (quite common in asthmatics and cystic fibrosis patients) (1) or noninvasive disease, such as bronchitis or sinusitis (2). On the other hand, invasive aspergillosis, in which the fungal spores invade the lung tissue or other internal organs, is a leading cause of death among patients receiving long-term immunosuppressive treatment for hematological malignancies or following organ transplantation (3). Further, invasive aspergillosis is also an emerging infection among critically ill patients with predisposing conditions, such as chronic obstructive pulmonary disease (COPD), cirrhosis of the liver, or nonhematologic cancer (4).

Antifungal therapy for invasive aspergillosis includes the azoles itraconazole (ITR), voriconazole (VOR), posaconazole (POS), and isavuconazole (ISA), as well as amphotericin B (AMB). Moreover, glucan synthesis inhibitors (echinocandins), particularly caspofungin (CAS), have been used and are approved as salvage therapy for invasive aspergillosis. VOR has been the drug of choice for the treatment of pulmonary invasive aspergillosis and is the first-line therapy recommended in recent treatment guidelines, with AMB, ISA, and CAS being recommended as alternatives or salvage therapy (5, 6).

The suboptimal outcomes with current treatment options have prompted investigators to evaluate alternative therapeutic approaches, such as combination therapy (7), and the emergence of azole resistance among Aspergillus species has resulted in combination therapy being recommended as first-line and salvage therapy in some circumstances (8). The emergence of azole resistance in Aspergillus fumigatus from clinical samples has been reported worldwide, although some geographical regions seem to have a higher incidence. Azole-resistant strains have been reported in patients with most clinical forms of Aspergillus infections, with an incidence of up to 8% being reported in cystic fibrosis patients, as an example (9).

To date, clinical trials evaluating combination therapy for invasive aspergillosis have provided relatively inconclusive and sometimes conflicting results. A study by Candoni et al. evaluating the use of a combination of CAS and VOR found an 80% positive response rate for primary therapy (10), while Raad et al. showed success rates of 27% and 31% for primary and salvage therapy, respectively (11). However, a large trial evaluating the combination of VOR and the echinocandin anidulafungin (AN) suggested a benefit of the combination versus VOR monotherapy in terms of survival, particularly in certain populations (7). A meta-analysis conducted by Panackal et al. compared the 12-week survival rate in invasive aspergillosis patients involved in 24 published studies, encompassing 629 patients treated with combination therapy (as primary therapy in 502 patients and salvage therapy in 127 patients) and 1,204 patients treated with monotherapy (as primary therapy in 973 patients and salvage therapy in 231 patients) (12). This analysis showed that salvage combination therapy increased the odds of 12-week survival by 80% over that with monotherapy, though the overall success rate between monotherapy and combination primary therapy was not significant (12).

In light of the increased incidence of azole-resistant Aspergillus strains and the unacceptably high mortality rate, despite advances in antifungal therapy, new antifungal agents and therapeutic approaches for invasive aspergillosis are needed. SCY-078, currently in development by Scynexis, Inc., is a novel glucan synthase inhibitor that has oral as well as intravenous formulations. SCY-078 is a semisynthetic derivative of enfumafungin that differs structurally from echinocandins but which shares the same mechanism of action. SCY-078 has been demonstrated to have *in vitro* and/or *in vivo* activity against Aspergillus species, including azole-resistant strains (13), as well as against Candida spp. including azole-resistant and most echinocandin-resistant strains (14) and Pneumocystis spp. (15) Additionally, SCY-078 showed high levels of activity against an uncommon CAS-resistant A. fumigatus strain (SCY-078 MIC, 0.12 μg/ml; CAS MIC, >16 μg/ml) (16). Further, SCY-078 demonstrated activity against ITR-resistant Aspergillus strains with a minimum effective concentration (MEC) range of 0.03 to 0.5 μg/ml (12). Thus, SCY-078 shows promise as a novel drug for the treatment and prevention of invasive aspergillosis.

In this study, we report the results of susceptibility testing of SCY-078, both alone and in combination, against clinical strains of Aspergillus taken from the fungal culture collection at the Center for Medical Mycology, Cleveland, OH.

## RESULTS

### SCY-078 singly demonstrated potent anti-Aspergillus activity.

The MEC range, MEC_50_, and MEC_90_ values of SCY-078 were <0.06 to 4, <0.06, and 0.125 μg/ml, respectively, against all Aspergillus isolates tested. In line with other glucan synthase inhibitors, the MIC values were 8-fold higher than the MEC values, with the MIC range, MIC_50_, and MIC_90_ being <0.06 to 32, 8, and 16 μg/ml, respectively, for the 50% inhibition endpoint. For the 100% inhibition endpoint, values were <0.06 to >32, 16, and 32 μg/ml, respectively. There was no difference in the MEC or MIC between the different species tested, with all values being within 2 dilutions (Table 1). The SCY-078 minimum fungicidal concentration for 90% of isolates tested (MFC_90_) was >32 μg/ml, indicating that the antifungal activity of this new drug is fungistatic against this panel of Aspergillus strains.

**TABLE 1:** Activity of SCY-078 against Aspergillus isolates determined by measurement of the MECs and MICs

Species	MEC (μg/ml)			MIC (μg/ml)					
				50% inhibition			100% inhibition		
	Range	50%	90%	Range	50%	90%	Range	50%	90%
A. flavus (*n* = 54)	<0.06 to 0.25	<0.06	<0.06	<0.06 to 32	16	16	<0.06 to 32	16	32
A. fumigatus (*n* = 134)	<0.06 to 4	<0.06	0.125	0.25 to 16	4	8	8 to >32	16	>32
A. niger (*n* = 27)	<0.06 to 0.5	<0.06	<0.06	<0.06 to 8	2	4	8 to 16	16	16
A. terreus (*n* = 72)	<0.06 to 0.125	<0.06	0.125	<0.06 to 16	8	8	1 to 16	16	16
Other Aspergillus spp. (*n* = 24)	<0.06 to 0.25	<0.06	<0.06	<0.06 to 8	2	8	8 to >32	16	32
A. glaucus (*n* = 5)	<0.06 to 0.125	ND^*a*^	ND	1 to 8	ND	ND	32	ND	ND
A. nidulans (*n* = 9)	<0.06 to 0.125	ND	ND	1 to 4	ND	ND	16 to >32	ND	ND
A. ustus (*n* = 1)	<0.06	ND	ND	8	ND	ND	16	ND	ND
A. versicolor (*n* = 8)	<0.06 to 0.25	ND	ND	<0.06 to 8	ND	ND	8 to 32	ND	ND
A. westerdijkiae (*n* = 1)	<0.06	ND	ND	1	ND	ND	8	ND	ND
All isolates (*n* = 311)	<0.06 to 4	<0.06	0.125	<0.06 to 32	8	16	<0.06 to >32	16	32

a ND, not determined (MIC_50_s and MIC_90_s were not determined due to the low number of isolates of these species tested).

The MICs of the comparator antifungals AMB and VOR were read at 100% inhibition, as indicated in the CLSI standard. The MIC range, MIC_50_, and MIC_90_ values for AMB were 0.5 to >16, 4, and 8 μg/ml, respectively. For VOR, the MIC range, MIC_50_, and MIC_90_ values were 0.06 to >8, 0.5, and 2 μg/ml, respectively (Table 2). The MFC_90_ was >8 μg/ml for both AMB and VOR.

**TABLE 2:** Activity of comparators AMB and VOR against Aspergillus isolates determined by measurement of the MICs^*a*^

Species	MIC (μg/ml)					
	Amphotericin B			Voriconazole		
	Range	50%	90%	Range	50%	90%
A. flavus (*n* = 54)	0.5 to 8	4	8	0.25 to 4	1	2
A. fumigatus (*n* = 134)	1 to >16	2	4	0.25 to >8	0.5	2
A. niger (*n* = 27)	1 to 4	1	2	0.25 to >8	1	2
A. terreus (*n* = 72)	1 to >16	8	8	0.06 to 1	0.5	1
Other Aspergillus spp. (*n* = 24)	1 to >16	4	16	0.125 to 8	0.5	2
A. glaucus (*n* = 5)	1 to 8	ND^*b*^	ND	0.25 to 2	ND	ND
A. nidulans (*n* = 9)	2 to >16	ND	ND	0.125 to 1	ND	ND
A. ustus (*n* = 1)	4	ND	ND	8	ND	ND
A. versicolor (*n* = 8)	1 to 16	ND	ND	<0.063 to 8	ND	ND
A. westerdijkiae (*n* = 1)	4	ND	ND	0.5	ND	ND
All isolates (*n* = 311)	0.5 to >16	4	8	0.6 to >8	0.5	2

a The MICs were read at the 100% inhibition endpoint.

b ND, not determined (MIC_50_s and MIC_90_s were not determined due to the low number of isolates of these species tested).

### SCY-078 tested in combination.

The combination of SCY-078 and VOR demonstrated synergistic activity against all four of the wild-type (WT) isolates tested (fractional inhibitory concentration index [FICI] range, 0.19 to 0.5). The combination of SCY-078 and VOR showed no interaction when tested against the azole-resistant A. fumigatus isolates (FICI range, 1 to 1.25; Table 3).

**TABLE 3:** Activity of SCY-078 plus VOR in combination against A. fumigatus strains by duplicate testing

MRL strain	MIC (μg/ml)				FICI	Interpretation^*a*^
	Drug tested alone		Drug in combination			
	SCY-078	VOR	SCY-078	VOR		
20438	8	1	0.125	0.25	0.27	S
	4	1	0.25	0.25	0.31	S
28378	8	0.5	0.5	0.125	0.31	S
	4	0.25	0.5	0.016	0.19	S
28382	8	0.5	0.5	0.125	0.31	S
	8	0.5	0.016	0.25	0.5	S
28401	8	2	0.25	0.5	0.28	S
	8	2	0.125	0.5	0.27	S
28383^*b*^	8	>16	0.031	>16	1	NI
	8	>16	0.031	>16	1	NI
28500^*b*^	4	>16	1	>16	1.25	NI
	8	>16	1	>16	1.13	NI

a S, synergistic interaction (FICI ≤ 0.5); NI, no interaction (0.5 < FICI ≤ 4.0).

b Azole-resistant strains.

The combination of SCY-078 and AMB demonstrated synergy against all WT strains tested, (FICI range, 0.13 to 0.25) but no interaction against the azole-resistant isolate A. fumigatus MRL 28383 (FICI = 1). Importantly, however, SCY-078 and AMB did demonstrate synergistic activity against the *cyp51* mutant strain A. fumigatus MRL 28500 (FICI = 0.28; Table 4).

**TABLE 4:** Activity of SCY-078 plus AMB in combination against A. fumigatus strains by duplicate testing

MRL strain	MIC (μg/ml)				FICI	Interpretation^*a*^
	Drug tested alone		Drug in combination			
	SCY-078	AMB	SCY-078	AMB		
20438	4	4	0.016	0.5	0.13	S
	4	4	0.016	0.5	0.13	S
28378	4	2	0.016	0.5	0.25	S
	4	2	0.016	0.5	0.25	S
28382	4	4	0.016	1	0.25	S
	8	4	0.063	0.5	0.13	S
28401	4	4	0.016	1	0.25	S
	8	4	0.031	0.5	0.13	S
28383^*b*^	4	4	0.016	4	1	NI
	4	2	0.125	2	1.03	NI
28500^*b*^	4	4	0.016	1	0.25	S
	4	4	0.125	1	0.28	S

a S, synergistic interaction (FICI ≤ 0.5); NI, no interaction (0.5 < FICI ≤ 4.0).

b Azole-resistant strains.

The combination of SCY-078 and ISA demonstrated synergistic activity against all four WT isolates tested (FICI range, 0.03 to 0.5). SCY-078 and ISA demonstrated no interaction against the two azole-resistant A. fumigatus strains (MRL 28500, a *cyp51* mutant, and MRL 28383; FICI range, 0.51 to 1.25) (Table 5).

**TABLE 5:** Activity of SCY-078 plus ISA in combination against A. fumigatus strains by duplicate testing

MRL strain	MIC (μg/ml)				FICI	Interpretation^*a*^
	Drug tested alone		Drug in combination			
	SCY-078	ISA	SCY-078	ISA		
20438	4	1	0.016	0.5	0.5	S
	4	1	0.016	0.5	0.5	S
28378	4	1	0.125	0.125	0.16	S
	4	1	0.125	0.25	0.28	S
28382	4	1	0.063	0.25	0.27	S
	8	>8	0.016	0.25	0.03	S
28401	4	1	0.25	0.25	0.31	S
	8	1	0.5	0.25	0.31	S
28383^*b*^	4	>8	0.031	4	0.51	NI
	4	>8	0.063	>8	1.02	NI
28500^*b*^	4	>8	0.125	>8	1.03	NI
	4	>8	1	>8	1.25	NI

a S, synergistic interaction (FICI ≤ 0.5); NI, no interaction (0.5 < FICI ≤ 4.0).

b Azole-resistant strains.

## DISCUSSION

High rates of morbidity and mortality persist in cases of invasive aspergillosis, despite the availability of new triazole and echinocandin antifungals. In response to the need for new effective antifungal agents to prevent or treat invasive aspergillosis, SCY-078, a novel glucan synthase inhibitor, is being developed as an alternative to existing therapy. As part of the preclinical regimen, we determined the *in vitro* activity of SCY-078, both alone and in combination with other antifungals, against a panel of clinical Aspergillus strains. Since other species, in addition to A. fumigatus, have been reported in invasive aspergillosis, we included strains of A. flavus, A. nidulans, A. niger, and A. terreus, among others. SCY-078 demonstrated potent fungistatic activity against all species tested, with 90% of all strains being inhibited at a concentration of ≤0.125 μg/ml when measured by determination of the minimum effective concentration (MEC); as with echinocandins, which have the same mechanism of action as SCY-078, this evidence of morphological damage may be better correlated with the clinical outcome than growth inhibition endpoints, though this remains to be proven through clinical use. MEC values were initially developed with CAS to reflect the fact that even though CAS was clinically active, the MIC as an endpoint did not reflect its potency. Therefore, the use of MEC for SCY-078 as well seems to be appropriate. SCY-078 MEC_90_ values were 4- and 6-fold lower than the MIC_90_ values of VOR and AMB, respectively. In light of the development of azole resistance in Aspergillus and the serious drug-drug interactions of azoles, along with the high toxicity rates associated with AMB, SCY-078 may prove a safer, more effective monotherapy for the treatment of Aspergillus infections.

Factors such as suboptimal outcomes (high mortality), drug-drug interactions, and azole resistance development have also led to an increased interest in the use of combination therapy to compensate for the shortcomings of monotherapy. Most of these combination studies have been based on the rationale of combining agents that have complementary mechanisms of action (17). Thus, recent studies have combined an azole, which targets cell activity with the alteration of 14-α-demethylase or the overexpression of drug efflux pumps (18), with an echinocandin, which blocks the production of 1,3-β-d-glucan, a fundamental component of the fungal cell wall (19). However, mixed success rates have been reported with the combined use of echinocandins and azoles. Because the mutations in the *FKS* genes responsible for echinocandin resistance are different from those associated with resistance to enfumafungin derivatives, such as SCY-078, this new drug may demonstrate different pharmacodynamics when used in combination.

Due to the difficulty of reading the MEC in the combination plates, we opted to evaluate the interactions between various drugs using the MIC as an endpoint. MEC endpoints rely on observing morphological aberrations. In the combination plates, such changes were difficult to observe due to masking by fungal growth. Our data show that the addition of SCY-078 to ISA or VOR resulted in synergistic activity against WT A. fumigatus strains. These results are especially encouraging, in that early treatment with either combination as soon as invasive aspergillosis is suspected and before azole susceptibility is established could reduce mortality rates. Predictably, though, these combinations did not have enhanced activity against azole-resistant strains. However, addition of SCY-078 to AMB resulted in synergy not only against all WT strains tested but also against the known *cyp51* mutant A. fumigatus strain. This suggests that combination therapy with SCY-078 and AMB may have greater utility in cases where azole resistance is suspected. Importantly, combination testing with SCY-078 showed no antagonism with ISA, VOR, or AMB, indicating that the addition of SCY-078 would not compromise the activity of concomitant azole or polyene therapy.

Overall, SCY-078 demonstrated potent activity against A. fumigatus and non-fumigatus Aspergillus isolates. This novel antifungal would be a much needed addition to the armamentarium available for the prevention and treatment of invasive aspergillosis. An important advantage would be the availability of SCY-078 in both oral and intravenous formulations, which would provide many more options for both monotherapy and combination therapy. Randomized clinical trials are needed in order to fully characterize the effectiveness of SCY-078.

## MATERIALS AND METHODS

### Effects of SCY-078 singly. (i) Isolates.

The isolates tested included 311 clinical Aspergillus strains and 1 reference A. flavus strain, ATCC 204304; the species included A. flavus (*n* = 54), A. fumigatus (*n* = 134), A. glaucus (*n* = 5), A. nidulans (*n* = 9), A. niger (*n* = 27), A. terreus (*n* = 72), A. ustus (*n* = 1), A. versicolor (*n* = 8), and A. westerdijkiae (*n* = 1). Seventeen strains of this test panel had previously demonstrated reduced susceptibility to AMB, VOR, and/or echinocandins.

### (ii) Antifungals.

The antifungals tested included SCY-078 (Scynexis, Inc., Jersey City, NJ) and the comparators VOR and AMB (Sigma-Aldrich, St. Louis, MO).

### (iii) Methods.

The MIC and minimum effective concentration (MEC) of SCY-078 and the MICs of the comparators were determined according to the CLSI M38-A2 susceptibility testing standard (20). Isolates were subcultured from a frozen stock to potato dextrose agar (PDA; Thermo Fisher Scientific, Waltham, MA) and incubated at 35°C until good conidiation was achieved. Spores were then harvested to sterile saline, and the inoculum was adjusted to approximately 0.4 × 10^4^ to 5 × 10^4^ CFU/ml in RPMI 1640 with MOPS (morpholinepropanesulfonic acid; US Biological Life Sciences, Salem, MA). Serial dilutions of SCY-078 and the comparators were prepared in RPMI 1640 and added to the wells of microtiter plates in 100-μl aliquots. The inoculum was then added in 100-μl amounts, and the plates were incubated at 35°C for 48 h. VOR and AMB visual endpoints were recorded at 100% inhibition, and SCY-078 visual endpoints were recorded at 50% and 100% inhibition, as was the MEC, which was the lowest concentration at which the growth in the wells exhibited small, rounded, compact hyphal forms but the growth in the control well showed hyphal forms, indicating gross morphological changes.

Minimum fungicidal concentrations (MFCs) were determined by subculturing the contents of visibly clear wells of the microtiter plates to PDA. Fungicidal activity was defined as a ≥99.9% reduction in the number of CFU per milliliter from the starting inoculum count. Static activity was defined as a <99.9% reduction (21).

### Effect of SCY-078 in combination. (i) Isolates.

For the combination assays, we tested six clinical strains of A. fumigatus, four wild-type (WT) and two azole-resistant strains, one of which (MRL 28500) is known to have an F46Y *cyp51* mutation.

### (ii) Antifungals.

ISA (Sigma-Aldrich, St. Louis, MO), VOR, and AMB were tested in combination with SCY-078.

### (iii) Methods.

Combination MIC testing of SCY-078 plus AMB, SCY-078 plus ISA, and SCY-078 plus VOR was performed using a checkerboard combination test method, a modification of the microdilution antifungal susceptibility test described above wherein two test compounds were combined in various concentrations to determine whether they have a synergistic, antagonistic, or no effect on the respective MIC values. Due to difficulty in reading the MEC endpoints in the combination plates visually, we used the MIC endpoint to capture the interaction between the drugs evaluated.

Antifungals were serially diluted 2-fold in RPMI 1640 to produce 11 concentrations each and combined in the wells of a microtiter plate. One row of serial dilutions of each individual drug alone was included. Comparison of the MICs of the individual drugs at 100% inhibition (VOR or AMB) or 50% inhibition (SCY-078) to the MIC of the combined agents was indicative of their relative efficacy.

Combination testing was reported according to the fractional inhibitory concentration index (FICI), which assigns a numerical value to the interaction of the two compounds and which is calculated as follows: (MIC of SCY-078 in combination/MIC of SCY-078 alone) + MIC of comparator in combination/MIC of comparator alone. The interpretation of the FICI for any combination of compounds is as follows: a synergistic interaction is an FICI of ≤0.5, no interaction is 0.5 < FICI ≤ 4.0, and an antagonistic interaction is an FICI of >4.0. This interpretation follows *Antimicrobial Agents and Chemotherapy* guidelines, which seek to encourage conservative interpretation of checkerboard combination data.
